# Biocontrol Rhizobacterium *Pseudomonas sp.* 23S Induces Systemic Resistance in Tomato (*Solanum lycopersicum* L.) Against Bacterial Canker *Clavibacter michiganensis* subsp. *michiganensis*

**DOI:** 10.3389/fmicb.2018.02119

**Published:** 2018-09-11

**Authors:** Yoko Takishita, Jean-Benoit Charron, Donald L. Smith

**Affiliations:** Department of Plant Science, McGill University, Montréal, QC, Canada

**Keywords:** tomato, *Pseudomonas*, *Clavibacter michiganensis* subsp. *michiganensis*, PGPR, biocontrol, induced systemic resistance

## Abstract

Tomato bacterial canker disease, caused by *Clavibacter michiganensis* subsp. *michiganensis* (*Cmm*) is a destructive disease and has been a serious concern for tomato industries worldwide. Previously, a rhizosphere isolated strain of *Pseudomonas sp.* 23S showed antagonistic activity toward *Cmm in vitro*. This *Pseudomonas sp*. 23S was characterized to explore the potential of this bacterium for its use in agriculture. *Pseudomonas sp*. 23S possesses ability to solubilize inorganic phosphorus, and to produce siderophores, indole acetic acid, and hydrogen cyanide. The strain also showed antagonistic activity against *Pseudomonas syringae* pv. *tomato* DC 3000. A plant assay indicated that *Pseudomonas sp*. 23S could promote growth of tomato seedlings. The potential of treating tomato plants with *Pseudomonas sp.* 23S to reduce the severity of tomato bacterial canker by inducing systemic resistance (ISR) was investigated using well characterized marker genes such as *PR1a* [salicylic acid (SA)], *PI2* [jasmonic acid (JA)], and *ACO* [ethylene (ET)]. Two-week-old tomato plants were treated with *Pseudomonas sp.* 23S by soil drench, and *Cmm* was inoculated into the stem by needle injection on 3, 5, or 7 days post drench. The results indicated that plants treated with *Pseudomonas sp*. 23S, 5 days prior to *Cmm* inoculation significantly delayed the progression of the disease. These plants, after 3 weeks from the date of *Cmm* inoculation, had significantly higher dry shoot and root weight, higher levels of carbon, nitrogen, phosphorus, and potassium in the leaf tissue, and the number of *Cmm* population in the stem was significantly lower for the plants treated with *Pseudomonas sp.* 23S. From the real-time quantitative PCR (qRT-PCR) analysis, the treatment with *Pseudomonas sp.* 23S alone was found to trigger a significant increase in the level of *PR1a* transcripts in tomato plants. When the plants were treated with *Pseudomonas sp.* 23S and inoculated with *Cmm*, the level of *PR1a* and *ACO* transcripts were increased, and this response was faster and greater as compared to plants inoculated with *Cmm* but not treated with *Pseudomonas sp.* 23S. Overall, the results suggested the involvement of SA signaling pathways for ISR induced by *Pseudomonas sp.* 23S.

## Introduction

Bacterial canker disease, caused by *Clavibacter michiganensis* subsp. *michiganensis* (*Cmm*) is one of the most destructive diseases in tomato (Gleason et al., 1993; de León et al., 2011). It has been reported in both greenhouse and field tomato production worldwide, and has caused substantial crop losses (Chang et al., 1992a; Hausbeck et al., 2000; de León et al., 2011). Once plants are infected by *Cmm*, initial marginal leaf necrosis symptoms widen and lead to wilting of all leaves while canker develops on the stem, and the whole plants can be stunted and severely wilted leading to death (de León et al., 2011; Sen et al., 2015). *Cmm* inoculum can originate from infected soils, seeds, transplants, tomato debris in soil, and operating tools and equipment. The bacteria can enter the plants through wounds and natural openings such as stomata and hydathodes after which they move to the xylem and multiply rapidly (Carlton et al., 1998; Gartemann et al., 2003; Sharabani et al., 2013). Farming practices such as tying, pruning, clipping, spraying and harvesting can cause a high level of secondary infection spread to nearby healthy plants via workers’ fingers and tools (Ark, 1944; Gleason et al., 1993). Despite the seriousness of this disease, no control methods have been found to be completely effective. As no *Cmm*-resistant seeds are commercially available, current control primarily relies on the use of pathogen-free certified seeds and transplants, good hygiene, disinfection of all tools, and crop rotations (Xu et al., 2015). Hence an effective control method for bacterial canker is urgently needed.

Use of plant growth-promoting rhizobacteria (PGPR) as biocontrol agents offers an ecological means to manage disease problems in agriculture. PGPR are rhizosphere free-living bacteria that colonize plant roots and have beneficial effects on plant growth (Kloepper and Schroth, 1978; Kloepper et al., 1989; Bouizgarne, 2013). The biocontrol ability of PGPR can be attributed to two general mechanisms: direct antagonism against pathogens or induction of systemic resistance throughout the plant. Production of antimicrobial compounds, such as antibiotic metabolites, and bacteriocin has been observed from many PGPR, and their inhibitory actions against pathogens contribute to reduction of plant diseases (Subramanian and Smith, 2015). In addition to direct suppressive effects on the pathogens, PGPR can trigger systemic resistance throughout the plant. PGPR-mediated induced systemic resistance (ISR) is often achieved by priming (Pieterse et al., 2014). Priming is characterized as potentiated activation of defense responses, which are subsequently induced upon pathogen attack, resulting in enhanced plant defense capacity (Conrath et al., 2006).

Although many ISR-inducing PGPR have been discovered, signaling and activation mechanisms of the ISR are not completely understood. The involvement of three plant hormones, salicylic acid (SA), jasmonic acid (JA), and ethylene (ET) have been well documented. Conventionally, SA is believed to be involved in systemic acquired resistance (SAR), which is induced by pathogens attack and follows induction of PR proteins. PGPR-mediated ISR is known to be dependent on JA and ET signaling; it is a SA-independent process and does not lead to induction of PR proteins (Van Loon and Bakker, 2005; Van Wees et al., 2008; Pieterse et al., 2014). As more research on ISR has been conducted, however, evidence of SA-dependent ISR has been observed for some PGPR (De Meyer et al., 1999; Tjamos et al., 2005; Schuhegger et al., 2006; Conn et al., 2008; Rudrappa et al., 2008; Niu et al., 2012).

Regarding tomato bacterial canker, several PGPR having antagonistic activities toward *Cmm* have been isolated and studied (Amkraz et al., 2010; Lanteigne et al., 2012; Deng et al., 2015; Aksoy et al., 2017). Among them, the induction of ISR was only reported for *Pseudomonas putida* (CKPp9; Aksoy et al., 2017). This ISR was accompanied by induction of significant amounts of phenolic compounds, which contributed to the disease reduction.

Our laboratory has been working to develop PGPR-based technologies for agriculture. As a part of our work on identifying new PGPR (Jung et al., 2014), we isolated a rhizobacterium that inhibited the growth of *Cmm in vitro* (**Supplementary Figure S1**). According to the 16S rRNA sequencing, this bacterium was identified as a strain of *Pseudomonas*, and is thus referred to here as *Pseudomonas sp*. 23S.

This study was conducted to achieve three objectives. The first objective was to characterize the newly isolated *Pseudomonas sp*. 23S for important PGPR traits. Our second objective was then to determine whether *Pseudomonas sp.* 23S induces ISR in tomato plants and reduces the disease severity specifically, bacterial canker, caused by *Cmm*. Not all PGPR possess ISR-inducing ability, and the question as to whether *Pseudomonas sp*. 23S, which has direct antagonistic activity against *Cmm*, is also able to induce ISR is meaningful to answer, because this could greatly enhance the use of this bacterium as a biocontol agent. Given that initial work reported in this paper did show that the bacterium was an ISR inducer, our final objective was then to determine whether treatment with *Pseudomonas sp.* 23S causes changes in the transcript levels of defense-related genes, specifically *PR1a*, *PI2* and *ACO*. Investigating the transcript levels of these three genes could help to determine the possible involvement of SA, JA and/or ET in the ISR response, and to understand the ISR signaling pathway used by *Pseudomonas sp*. 23S specific to this biotic stress.

## Materials and Methods

### Bacterial Growth Condition

*Pseudomonas sp.* 23S was grown in Nutrient Broth (NB, Difco; 8 g L^−1^) media at 28°C, at 100 rpm. *C. michiganensis* subsp. *michiganensis* strain 930 (*Cmm*) was provided by Agriculture, Pecheries et Alimentation, Quebec. *Cmm* was grown in NB media at 28°C, at 150 rpm. Both bacteria were maintained as a glycerol stock in −80°C.

### *In vitro* Assay for General PGPR Traits

*Pseudomonas sp.* 23S was assessed for important PGPR traits: (i) phosphorous solubilization; (ii) siderophore production; (iii) hydrogen cyanide production; (iv) indole acetic acid production, and; (v) antagonistic activity against an important phytopathogenic bacterium. The abilities to solubilize phosphorous and to produce indole acetic acid were investigated since these traits improve plant growth. Production of siderophores and hydrogen cyanide, both of which can suppress phytopathogenic bacteria, was studied for the biocontrol traits. Phosphorous solubilization was studied using Pikovskaya medium (PVK; Pikovskaya, 1948) and the National Botanical Research Institute’s phosphate growth medium (NBRIP; Nautiyal, 1999). The two types of plates were used to corroborate the results since the PVK plate could sometime give variable results (Nautiyal, 1999). The Fiske and Subbarow method (Fiske and Subbarow, 1925) was applied for quantitative evaluation. Siderophore production was studied using the chrome azurol S (CAS) assay developed by Alexander and Zuberer (1991). For quantitative assessment, percent siderophore production was calculated by using the following formula:

% siderophore production=Ar−As/Ar×100

Where, Ar represents the absorbance of reference (CAS assay solution plus growth medium) at 630 nm and As represents the sample (CAS assay solution plus bacterial supernatant) at 630 nm (Schwyn and Neilands, 1987; Alexander and Zuberer, 1991; Ghosh et al., 2015).

For the hydrogen cyanide, *Pseudomonas sp*. 23S was grown in Kings B medium (per L of distilled H_2_O: proteose peptone No.3 20 g, glycerol 10 mL, K_2_HPO_4_ 1.5 g, MgSO_4_ 1.5 g), in which glycerin serves as a precursor molecule (Knowles, 1976; Askeland and Morrison, 1983; Schippers et al., 1990). Indole acetic acid production was evaluated as described by Deaker et al. (2011). In the NB medium where *Pseudomonas sp.* 23S was grown, DL-tryptophan (TM 7425 Sigma) was added to serve as a precursor of IAA, at two concentrations 0.5 g L^−1^ or 1.0 g L^−1^. Antagonistic activity of *Pseudomonas sp*. 23S was assessed against *Pseudomonas syringae* pv. *tomato* DC3000 (provided by Dr. Diane Cuppels, AAFC, London). *Pseudomonas syringe* pv. *tomato* DC3000 was grown in Kings B media and 100 μL of 2-day-old culture was spread on Kings B agar plates. A sterile filter-paper disk, with 10 μL of *Pseudomonas sp*. 23S culture was placed on each pathogen inoculated plate, and the plates was sealed with parafilm and incubated for at least 2 days at 28°C, to observe development of inhibition zones.

### Seedling Assay for Plant Growth Promotion

Tomato seeds (Bush Beefsteak 351; Stroke Seeds Inc., Thorold, ON, Canada) were surface-sterilized by soaking in 3% (v/v) hypochlorite solution for 3 min, washing thoroughly with water, and drying overnight. The seeds were sown in pots (7.5 mm diameter; 2 seeds pot^−1^) filled with a mix of sand and turface (5:5). The pots were washed with bleach, and a mix of sand and turface was autoclaved prior to use. The seedlings were thinned to leave 1 plant pot^−1^ after emergence. After 7 days from the day of seeding, 50 mL suspension of *Pseudomonas sp*. 23S (10^8^ cfu mL^−1^ in 10 mM MgSO_4_) was applied to each pot as a soil drench. For the control treatment, 50 mL of 10 mM MgSO_4_ was applied to each pot. The seedlings were grown in a growth chamber with a 14/10 h photoperiod and a 25/23°C day/night temperature. Sterilized water was applied as needed. After 3 and 7 days from *Pseudomonas sp.* 23S treatment (10 and 14-day-old seedlings), the population of *Pseudomonas sp.* 23S around the roots was enumerated. For this, the seedling was removed from the soil, shaken to dislodge the soil as much as possible, and the root was cut from the plant. The root was then ground with a mortar and pestle in 500 μL of 10 mM MgSO_4_. The solution was centrifuged (10,000 rpm, 1 min) to remove the debris, and 100 μL of its serial dilution was plated on *Pseudomonas* Isolation Agar plates (PIA; Difco). The plates were incubated overnight at 28°C, after which the number of colonies formed was counted. Eleven days after *Pseudomonas sp.* 23S treatment (18-day-old seedlings), the whole plants were harvested. The shoots were dried in an incubator for 2 days at 60°C and shoot dry weight was determined. The roots were first scanned (Modified Epson Expression 10000XL, Regent Instruments Inc., Quebec, QC, Canada) at 400 dots per inch (dpi) resolution and then, images were analyzed by using WinRHIZO software (Reagent Instruments Inc.) to study morphological features and later used for determination of root dry weight. There were five biological replicates per treatment at each time point for the population study, and there were seven biological replicates for the dry weight study. The experiment was conducted twice. A student’s *t*-test was applied to determine significant differences between control and bacterial treatments.

### Effects of *Pseudomonas* sp. 23S on Bacterial Canker

Tomato seeds were surface-sterilized as described above. The seeds were sown into pots (13 mm diameter) filled with agromix (G10). The plants were grown in a plant growth chamber under the following conditions: 16/8 h of photoperiod, 25/20°C of day/night temperature, and 65% of relative humidity; they were watered daily. Once true leaves emerged, half-strength Hoagland solution was provided once a week (Hoagland and Arnon, 1950; PlantMedia #30630037-5). The experiment was organized following a factorial design with two levels of *Pseudomonas sp.* 23S treatments (+ and −), and two levels of *Cmm* inoculation (+ and −). Treatments consisted of: (1) *Cont*, without *Pseudomonas sp.* 23S treatment, and *Cmm* inoculation (negative control); (2) *Pse*, treated with *Pseudomonas sp.* 23S; (3) *Cmm*, without *Pseudomonas sp.* 23S treatment, and inoculated with *Cmm*; and, (4) *P+C*, treated with *Pseudomonas sp.* 23S, and inoculated with *Cmm*. After 2 weeks from sowing, the plants were treated with *Pseudomonas sp.* 23S. Each plant received 100 mL of the *Pseudomonas sp.* 23S cells suspended in 10 mM MgSO_4_ (approximately 10^8^ cfu mL^−1^) for *Pse* and *P+C* treatments and 100 mL of 10 mM MgSO_4_ for *Cont* and *Cmm* treatments. The *Cmm* inoculation was conducted on one of the 4 days, after 1, 3, 5, and 7 days from the date of *Pseudomonas sp.* 23S treatment (corresponding to 15, 17, 19, and 21 day-old plants); 10 μL of *Cmm* cells suspended in 10 mM MgSO_4_ (approximately 10^8^ cfu mL^−1^) for *Cmm* and *P+C*, or 10 μL of 10 mM MgSO_4_ (approximately 10^8^ cfu mL^−1^) was inoculated by injecting into the main stem, where the cotyledon emerged, in each plant using a syringe (31 gauge needle, Thermo Scientific #3170513). The number of wilted leaves was counted for monitoring the disease progression. Area under disease progress curve (AUDPC) was calculated based on the formula Σ[(d_i+1_ + d_i_)/2](t_i+1_ - t_i_), where d_i+1_ and d_i_ are the percentage of wilted leaves and t_i+1_ and t_i_ are the days after *Cmm* inoculation (Xu et al., 2012). After 3 weeks from the date of *Cmm* inoculation, plants were harvested, and shoots (leaves and stems) and root dry weight, plant height, and leaf areas were measured. For each plant, a stem piece (approximately 1 cm) was sampled from 2-cm above the *Cmm* inoculation site in order to evaluate the *Cmm* population (number of cells per gram of tissue). The stem piece was weighed and ground with 1 mL of 10 mM MgSO_4_ using mortar and pestle. The extract was centrifuged at 10,000 rpm for 1 min, and 10-fold serial dilutions of the supernatant were plated on *Cmm*-selective agar plates (Ftayeh et al., 2011). The number of *Cmm* colonies were enumerated after 3–4 days after incubation at 28°C. Dried shoot tissues were ground to fine powder with mortar and pestle and used to analyze carbon (C), nitrogen (N), phosphorus (P), and potassium (K) contents. An elemental analyzer (Model NC2500; CE Instruments, Milan, Italy) was used for the C and N analysis. For the P and K analysis, a flow injection analyzer and atomic absorption spectrophotometer were used, respectively (Parkinson and Allen, 1975). First, the tissues were digested in sulfuric acid and peroxide with the addition of catalysts (lithium and selenium) at 340°C for approximately 3 h. The content was diluted to 100 mL and used for the flow injection analyzer. Phosphorous was measured colorimetrically at 880 nm following a complexation with ammonium molybdate (Lachat Instruments QuikChem Method 13-115-01-1-B, 6645 West Mill Road, Milwaukee, WI, United States). Potassium was read on a 10-fold diluted subsample (from the same diluted sample used for the P analysis) by emission on a Varian 220FS (now part of Agilent) atomic absorption spectrophotometer. Seven plants were sampled for each inoculation time (1, 3, 5, and 7 days) per treatment, and the experiment was repeated twice. Since the results from these two experiments were comparable, they were combined and presented in this paper.

### Quantitative Real-time PCR (qRT-PCR) Analysis

Two analyses were performed to study: (1) the effects of *Pseudomonas sp.* 23S on defense-related genes of tomato plants, and (2) the effects of *Pseudomonas* *sp.* 23S on defense-related genes of tomato plants that are infected with bacterial canker by *Cmm*. For the first analysis, the plants were treated with *Pseudomonas sp.* 23S with cells suspended in 10 mM MgSO_4_ (approximately 10^8^ cfu mL^−1^), and applied as a soil drench, 2 weeks after sowing. For control plants, 100 mL of 10 mM MgSO_4_ was applied to each plant in the same manner. At 1, 3, 5, and 7 days after the date of *Pseudomonas sp.* 23S application, the shoot was harvested (biomass pooled for each four replicate plants), flash-frozen with liquid nitrogen, and stored in −80°C for subsequent real-time quantitative PCR (qRT-PCR) analysis. For the second analysis, tomato plants were treated in the same manner as described for the first analysis, with a 5 day-time interval between *Pseudomonas* *sp*. 23S application and *Cmm* inoculation. At 1, 3, 5, and 7 days after the date of *Cmm* inoculation, the shoot was harvested (pooled for the four replicate plants), flash-frozen with liquid nitrogen, and stored at −80°C for subsequent qRT-PCR analysis. For both situations, the plants were grown under growth chamber condition as described above, and the experiment was conducted three times with independent biological replicates. For the qRT-PCR-analysis, total RNA from tomato leaves was extracted using TRIzol Reagent (Thermo Fisher Scientific catalog#: 15596026), and the RNAs were treated with DNase I (Ambion^TM^ DNaseI, Thermo Fisher Scientific catalog#: AM2222) according to the manufacturer’s instructions. The integrity of the extracted RNA was checked on agarose gel electrophoresis, and its purity and concentration were assessed by a ND-1000 spectrophotometer (NanoDrop). Complementary DNA (cDNA) was synthesized using an iScript Advanced cDNA Synthesis Kit (Bio-Rad, catalog#: 1725037), following the manufacturer’s instructions. The cDNA was diluted to 400 ng uL^−1^ and stored in −20°C for qPCR. Primer sequences, linear equations, correlation coefficients (R^2^), and reaction efficiencies for each gene used in this study are provided in **Table 1**. The qPCR was conducted on a CFX Connect Real Time System (Bio-Rad) with Green-2-Go qPCR Mastermix (Biobasic, catalog#: QPCR004-S), using the cycling program of: 95°C for 10 min for the enzyme activation step, 95°C for 15 s for the initial denaturation step, 60°C for 60 s for annealing and extension, repeated for 40 cycles (*PR1*: 52°C; *PI2*: 56°C; *ACO*: 52°C: *GAPDH*: 56.4°C). Each plate consisted of three technical replicates from the three independent biological replicates. The Ct value obtained was normalized against the housekeeping gene *GAPDH*, and the relative gene expression (fold change) was calculated using 2^−ΔΔCT^ method (Livak and Schmittgen, 2001).

**Table 1:** List of tomato genes used for the gene expression study.

ID	Target gene	Primer sequence (5′ to 3′)	Linear equation	Correlation coefficient (R^2^)	PCR efficiency
M69247	*Pathogenesis related protein* (*PR1a*)^1^	GTGGGATCGGATTGATATCCT CCTAAGCCACGATACCATGAA	*Y* = 3.394X + 27.172	0.991	98.3
X94946	*Proteinase inhibitor (PI2*)^2^	AATTATCCATCATGGCTGTTCAC CCTTTTTGGATCAGATTCTCCTT	*Y* = 3.471X + 25.992	0.997	97.1
AB013101	*1-aminocyclopropane-1-carboxylix acid oxidase* (*ACO*)^3^	AAGATGGCACTAGGATGTCAATAG TCCTCTTCTGTCTTCTCAATCAAC	*Y* = 3.545X + 18.479	0.985	94.1
U97257	*GAPDH^4^*	CTGGTGCTGACTTCGTTGTTG GCTCTGGCTTGTATTCATTCTCG	*Y* = 3.362X + 17.064	0.993	91.5

*^1^López-Ráez et al. (2010) and Martínez-Medina et al. (2013); ^2^Song et al. (2015); ^3^Yim et al. (2014); ^4^Chalupowicz et al. (2010).*

## Results

### *Pseudomonas* *sp.* 23S Showed Characteristic PGPR Traits

To explore its potential, *Pseudomonas* *sp.* 23S was studied for general PGPR traits: phosphorous (P) solubilization, production of siderophores, of hydrogen cyanide, of indole acetic acid, and antagonistic activity against phytopathogens. Pikovskaya (PVK) and National Botanical Research Institute’s phosphate growth medium (NBRIP) were used in P solubilization assay. In both PVK and NBRIP plates, the *Pseudomonas sp.* 23S inoculation resulted in a halo around the disk (**Supplementary Figure S2.1**; approximately 1 mm for each plate). The quantitative assay showed that *Pseudomonas sp.* 23S solubilized 5.84 μg mL^−1^ (±0.85) of inorganic phosphorus. The chrome azurol S (CAS) assay was performed to assess siderophore production. The *Pseudomonas sp.* 23S inoculation changed the blue color of CAS plates to orange (**Supplementary Figure S2.2**; size of halo was 4 mm). The quantitative assay showed that its production is 50.7% (±3.38). Hydrogen cyanide (HCN) production was examined by change in color soaked in picric acid. The negative control plate, where media had been applied, was bright yellow (**Supplementary Figure S2.3a**) whereas the positive control plate, where HCN-positive bacterium had been applied, was bright orange (**Supplementary Figure S2.3c**). *Pseudomonas sp.* 23S containing plate was neither this bright yellow nor bright orange, but rather a light orange color (**Supplementary Figure S2.3b**). To determine whether *Pseudomonas sp.* 23S could produce indole acetic acid (IAA), two concentrations of tryptophan, 0.5 g mL^−1^ and 1.0 g L^−1^, were used as a precursor for production of IAA. The quantitative assay indicated that *Pseudomonas sp.* 23S produced 1.96 μg IAA mL^−1^ (±0.09) at 0.5 g L^−1^ tryptophan and 2.72 μg IAA mL^−1^ (±0.07) at 1.0 g L^−1^ tryptophan. Antagonistic activity of *Pseudomonas sp.* 23S against *Pseudomonas syringe* pv. *tomato* DC 3000 was assessed. *Pseudomonas sp.* 23S inhibited the growth of the *Pseudomonas syringe* pv. *tomato* DC 3000, as indicated by inhibition zones around the disk (**Supplementary Figure S2.4**; the size of the inhibition zone was 4 mm).

### *Pseudomonas sp*. 23S Promoted Growth of Tomato Seedlings

**FIGURE 1:**
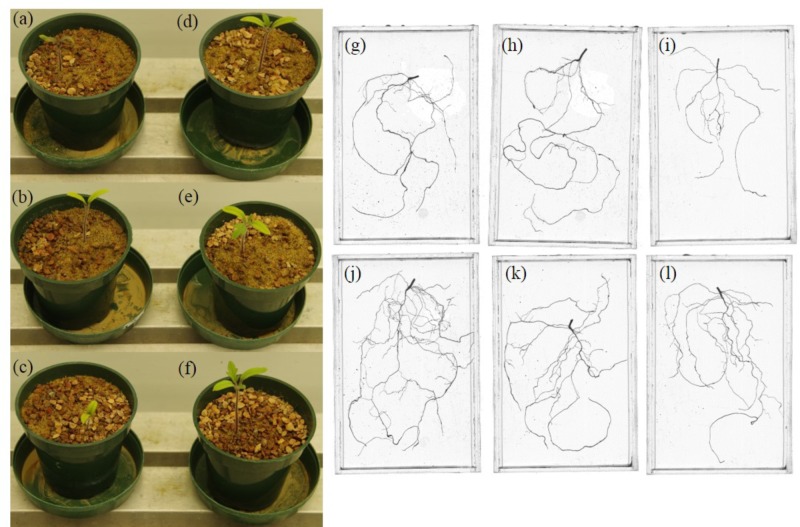
*Pseudomonas sp.* 23S promoted the growth of tomato seedlings. Seedlings from control **(a–c)** and from *Pseudomonas sp.* 23S treatment **(d–f)**, and roots from control **(g–i)** and from *Pseudomonas sp.* 23S treatment **(j–l)**.

*Pseudomonas sp.* 23S was applied as a soil drench and its effect on growth was examined for tomato seedlings. As **Figure 1** shows, *Pseudomonas sp*. 23S treated seedlings were visually bigger, and the dry weights of their shoots and roots were significantly higher (approximately 47% increase; **Figures 1d–f**) than those of control seedlings (**Figures 1a–c**). Roots of *Pseudomonas sp.* 23S treated seedlings appeared finer and longer (**Figures 1j–l**) than the roots of control seedlings (**Figures 1g–i**). Based on the root scanning analysis, root length, volume, and surface area of the *Pseudomonas* sp. 23S treated seedlings were significantly greater than those of control seedlings (**Table 2**). When the viable number of *Pseudomonas sp.* 23S cells around the root was enumerated, it was 10^5.08^ (±0.12) and 10^5.49^ (±0.14) colony forming units per seedling after 3 and 7 days, respectively.

**Table 2:** *Pseudomonas sp.* 23S treatment increased dry weight of shoots and roots, and improved root length, volume and surface area.

	Control	*Pseudomonas sp*. 23S
Dry shoot weight (mg)	8.81 ± 0.58	13.0 ± 0.78^∗^
Dry root weight (mg)	4.91 ± 0.48	7.18 ± 0.70^∗^
Root length (cm)	92.14 ± 5.83	123.18 ± 8.91^∗^
Root volume (mm^3^)	72.40 ± 4.59	97.27 ± 6.01^∗^
Root diameter (mm)	0.32 ± 0.0054	0.32 ± 0.0032
Root surface area (cm^2^)	9.13 ± 0.55	12.26 ± 0.82^∗^

*Data represented as mean ± SE (n = 15). An asterisk indicates significant difference from the control after the student’s t-test (p = 0.05).*

### *Pseudomonas sp.* 23S Alleviated Bacterial Canker by ISR

**FIGURE 2:**
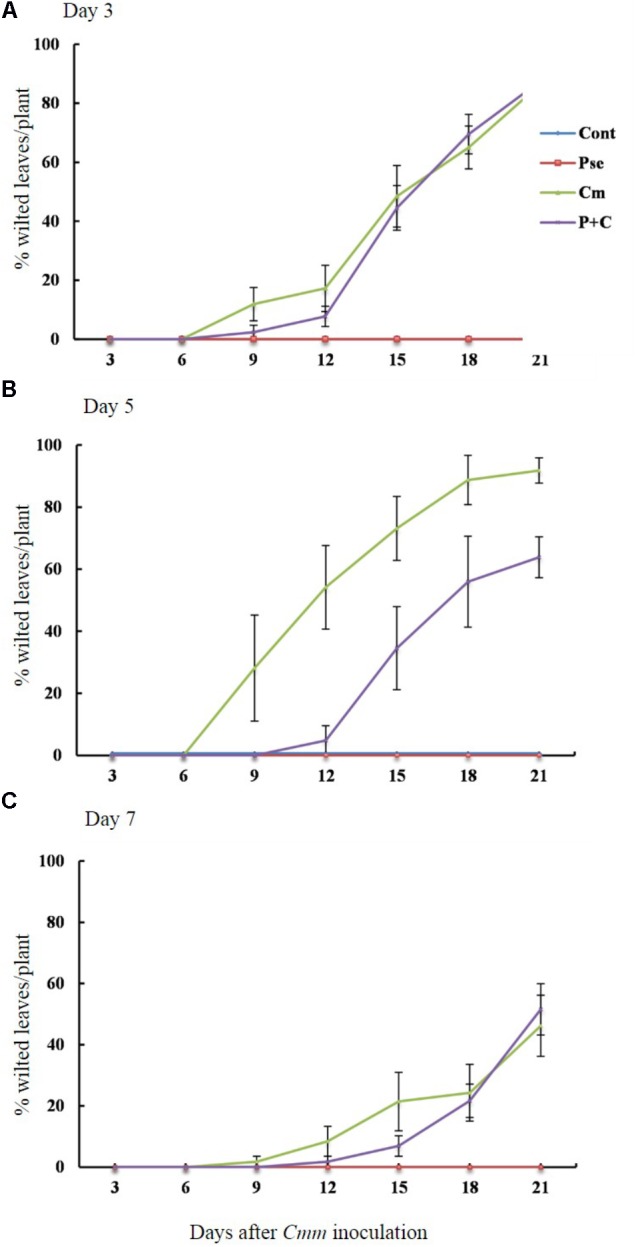
*Pseudomonas sp.* 23S treatment, 5 days prior to *Cmm* inoculation, delayed progression of bacterial canker. Two-week-old tomato plants were treated with *Pseudomonas sp.* 23S by soil drench, and after 3 days **(A)**, 5 days **(B)**, or 7 days **(C)**, *Cmm* was inoculated in the main stem by needle injection. The number of wilted leaves was counted every 3 days for 3 weeks. Treatments are: *Cont*, control; *Pse*, treated with *Pseudomonas sp.* 23S; *Cmm*, inoculated with *Cmm*; and, *P+C*, treated with *Pseudomonas sp.* 23S, and inoculated with *Cmm. Cont* and *Pse* treatments showed no disease symptom throughout the experiment (*n* = 14, *p* = 0.05).

The effects of *Pseudomonas sp.* 23S on the disease progression were studied for tomato plants infected with *Cmm* when the time interval between *Pseudomonas sp.* 23S application and *Cmm* inoculation dates were 3, 5, and 7 days (**Figures 2A–C**). The percentage of wilted leaves increased over time under 3-day-interval and reached more than 80% at 21 days post-*Cmm* inoculation (**Figure 2A**). Most of these plants were dead; the main stems were broken at the site where the *Cmm* was inoculated. When the interval was 5 days, the *Cmm* treatment resulted in a disease progression similar to the *Cmm* treatment for the 3-day-interval. However, the disease progression for the *P+C* treatment was significantly slower, and the percentage of wilted leaves was about 60% at 21 days post-*Cmm* inoculation (**Figure 2B**). For these plants, the main stems were not broken, the symptom observed in most severely infected plants following the *Cmm* treatment (**Supplementary Figure S3**). Under the 7-day-interval, the disease progression was slower than the disease progression at 3- and 5-day intervals. The percentage of wilted leaves was also smaller at 21 days post-*Cmm* inoculation, less than 60% for the *Cmm* treatment (**Figure 2C**). Under the 5-day interval, the AUDPC from the *P+C* treatment was significantly lower than that from the *Cmm* treatment (239 for *P+C* treatment and 576 for *Cmm* treatment; **Supplementary Figure S4**). There were no disease symptoms observed for the *Cont* and *Pse* treatments at any time.

**FIGURE 3:**
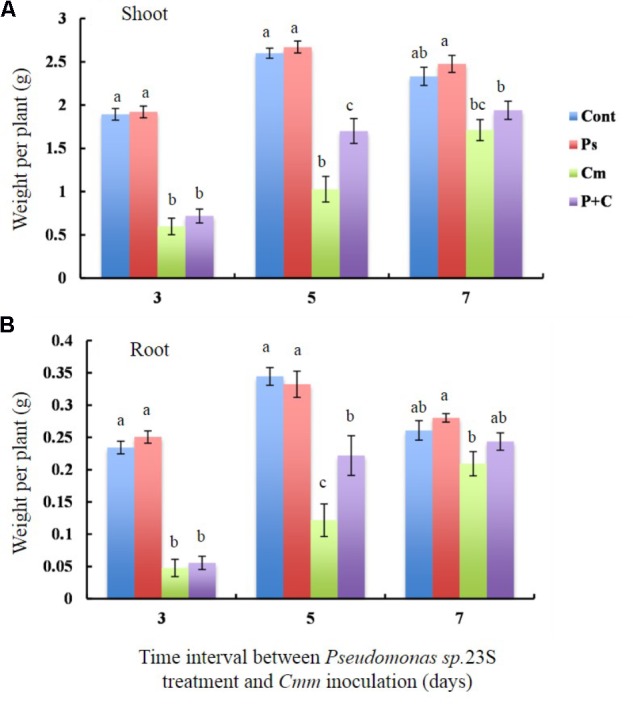
*Pseudomonas sp.* 23S treatment, 5 days prior to *Cmm* inoculation, increased the weights of shoots and roots. Two-week-old tomato plants were treated with *Pseudomonas sp.* 23S (or 10 mM MgSO_4_) by soil drench, and after 3, 5, or 7 days, *Cmm* (or 10 mM MgSO_4_) was inoculated into the main stem by needle injection. The plants were harvested after 3 weeks and sampled for: **(A)** weight of dry shoots and **(B)** weight of dry roots. Error bars indicate standard error of the mean. Association with different letters indicates statistical significance based on ANOVA followed by Tukey’s multiple comparison test. Treatments are: *Cont*, control; *Pse*, treated with *Pseudomonas sp.* 23S; *Cmm*, inoculated with *Cmm*; and, *P+C*, treated with *Pseudomonas sp.* 23S, and inoculated with *Cmm* (*n* = 14, *p* = 0.05).

To determine whether *Pseudomonas sp*. 23S treatment results in improvement of plant biomass, the dry weights of shoots and roots for the tomato plants grown under our experimental conditions were measured (**Figures 3A,B**). The shoot dry weights of the *Cont* and *Pse* treatments were not significantly different for any of the intervals (**Figure 3A**). With an interval of 3 and 5 days, the shoot dry weights of the *Cont* and *Pse* treatments were significantly different from those of the *Cmm* and *P+C* treatments while they were not significantly different with an interval of 7 days. Under the 3 and 7-days intervals, the shoot dry weights of the *Cmm* and *P+C* treatments were not significantly different. On the other hand, the shoot dry weight was greater for the *P+C* treatments than *Cmm* treatments under the 5-day-interval. Similar trends were found for the root dry weight, plant height, and leaf areas (**Figure 3B** and **Supplementary Figure S5**).

At the harvest (21 days post-*Cmm* inoculation), a 1-cm-length stem piece above the inoculation site was taken and used for counting the colony forming units (cfu) of *Cmm* presence (**Table 3**). The number of cfu for the *P*+*C* treatment was significantly lower than that for the *Cmm* treatment when the interval was 5 days, while no difference was detected when the intervals were 3 and 7 days.

**Table 3:** *Pseudomonas sp.* 23S treatment, 5-day prior to *Cmm* inoculation, reduced the *Cmm* population in the stem.

	Day 3	Day 5	Day 7
*Cmm*^a^	6.84 ± 0.17	6.57 ± 0.15	6.11 ± 0.06
*P+C*^b^	7.19 ± 0.08	6.16 ± 0.16^∗^	6.21 ± 0.16

*Two-week-old tomato plants were treated with Pseudomonas sp. 23S (or 10 mM MgSO_4_) by soil drench, and after 3, 5, or 7 days of Cmm (or 10 mM MgSO_4_) inoculation into the main stem by needle injection. After 3 weeks, 1-cm piece of stem (2 cm above the inoculation site) was sampled for enumeration of Cmm cells. The values represent log_10_ of the number of Cmm colony forming units (cfu). ^a^Cmm, inoculated with Cmm. ^b^P+C, treated with Pseudomonas sp. 23S, and inoculated with Cmm. Asterisk indicates statistical significance between the Cmm and P+C treatments based on the student’s t-test (n = 14, p = 0.05).*

**FIGURE 4:**
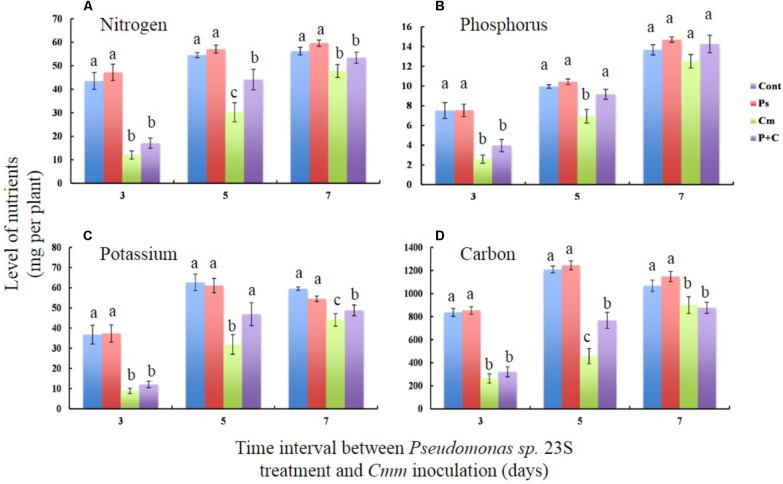
*Pseudomonas sp.* 23S treatment, 5 days prior to *Cmm* inoculation, improved nutrient levels of leaf tissue. Two-week-old tomato plants were treated with *Pseudomonas sp.* 23S (or 10 mM MgSO_4_) by soil drench, and after 3, 5, or 7 days, *Cmm* (or 10 mM MgSO_4_) was inoculated into the main stem by needle injection. The plants were harvested after 3 weeks. Nutrients analyzed are **(A)** Nitrogen, **(B)** Phosphorus, **(C)** Potassium, and **(D)** Carbon. Association with different letters indicates statistical significance based on ANOVA followed by Tukey’s multiple comparison test. Treatments are: *Cont*, control; *Pse*, treated with *Pseudomonas sp.* 23S; *Cmm*, inoculated with *Cmm*; and, *P+C*, treated with *Pseudomonas sp.* 23S, and inoculated with *Cmm* (*n* = 14, *p* = 0.05).

Nutrient levels of shoots, specifically nitrogen (N), phosphorus (P), potassium (K), and carbon (C) were measured to study the effects of the *Pseudomonas sp.* 23S treatment (**Figures 4A–D**). For all the nutrients that were measured, the levels were not significantly different between the *Cont* and *Pse* treatments under any of the interval times. On the other hand, the levels of the nutrients from the *Cmm* and *P+C* treatments were significantly lower than those of the *Cont* and *Pse* treatments. With an interval of 5 days, the levels of N, P, K, and C for the *P+C* treatment were significantly higher than those of the *Cmm* treatment. In addition, the nutrient levels of P and K for the *P+C* treatment were not different from those of the *Cont* treatment (**Figures 4B,C**). Under the 7-day-interval, the levels of the *Cmm* treatment tended to be lower but overall, the levels for all the nutrients were not very different among the treatments (**Figures 4A–D**).

### *Pseudomonas sp.* 23S Treatment Increases the Transcript Level of *PR1a*

**FIGURE 5:**
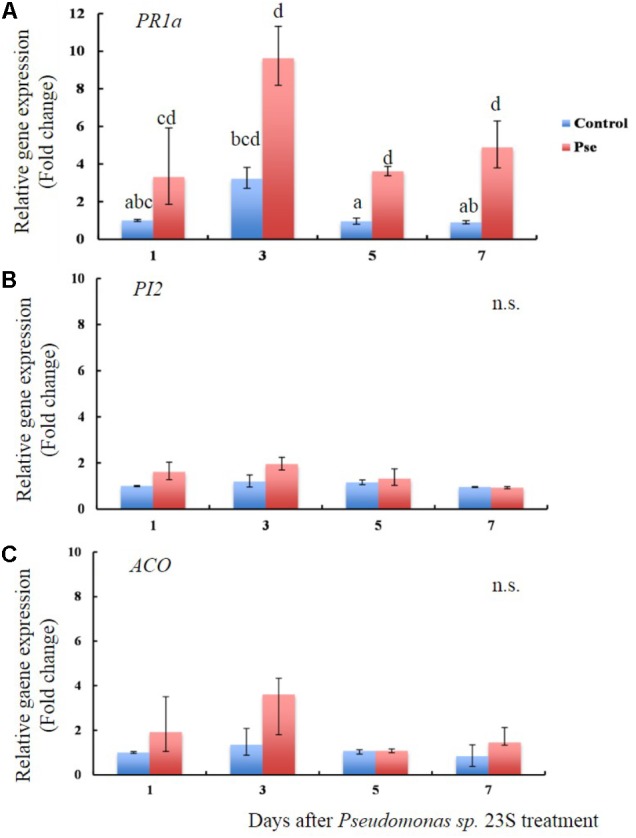
*Pseudomonas sp.* 23S treatment induced an increase in the transcript level of *PR1a.* Two-week old tomato plants were treated with *Pseudomonas sp.* 23S (Pse) or 10 mM MgSO_4_ (Control) and after 1, 3, 5, and 7 days, the shoots were harvested and used for RNA extraction and real-time qPCR. Genes analyzed are **(A)** *PR1a*, **(B)** *PI2*, and **(C)** *ACO*. Error bars indicate standard error of the mean. Association with different letters indicates statistical significance based on ANOVA followed by Tukey’s multiple comparison test (*p* = 0.05).

The transcript levels of *PR1a*, *PI2*, and *ACO* were studied in tomato plants 1, 3, 5, and 7 days after soil drench treatment with *Pseudomonas sp.* 23S (**Figures 5A–C**). The transcript levels of *PR1a*, a marker gene of salicylic acid activity were higher for the *Pseudomonas sp.* 23S treatment as compared with those of the Control treatment at all the time points (**Figure 5A**). Its transcript abundance reached highest at day 3 (10-fold), then diminished at days 5 (fourfold) and 7 (fivefold). The transcript levels of *PI2* and *ACO* were not different between Control and *Pseudomonas sp.* 23S treatments at any of the time points (**Figures 5B,C**).

### *Pseudomonas sp.* 23S Prior to *Cmm* Inoculation Caused Faster and Greater Accumulation of *PR1a* and *ACO* Transcripts

**FIGURE 6:**
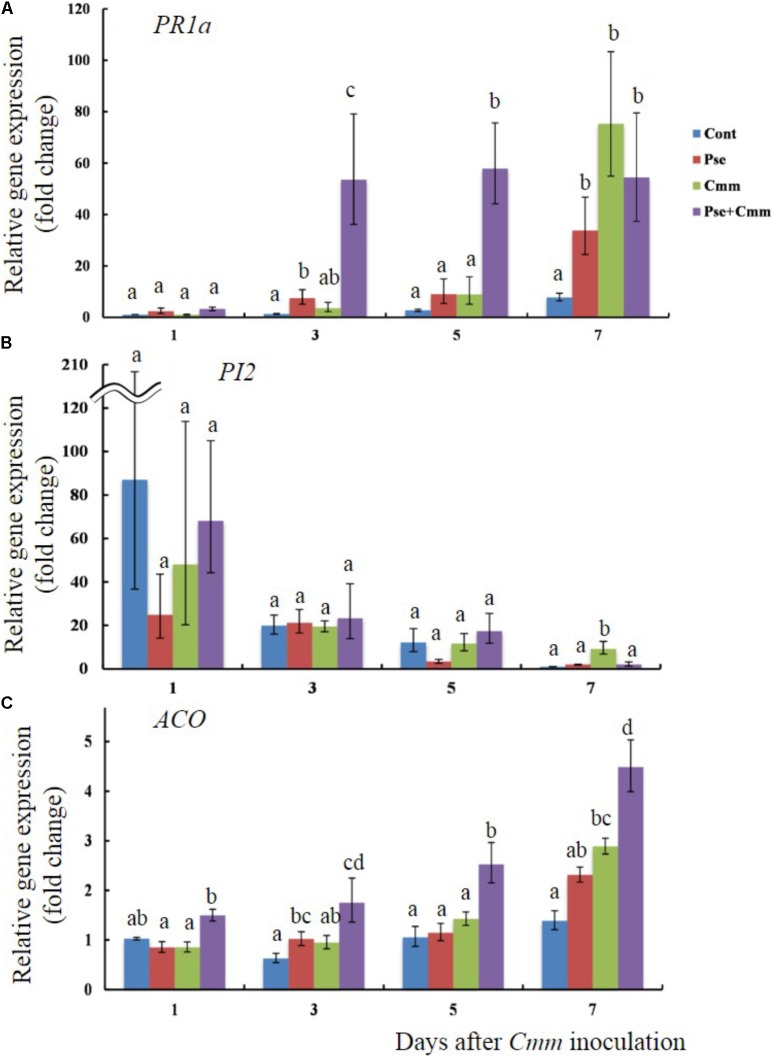
*Pseudomonas sp.* 23S treatment, 5 days prior to *Cmm* inoculation, induced faster and greater response in transcript levels of *PR1a* and *ACO.* Two-week old tomato plants were treated with *Pseudomonas sp.* 23S (or 10 mM MgSO_4_) and after 5 days, *Cmm* (or 10 mM MgSO_4_) was inoculated into the stem by needle injection: *Cont*, control; *Pse*, treated with *Pseudomonas sp.* 23S; *Cmm*, inoculated with *Cmm;* and, *P+C*, treated with *Pseudomonas sp.* 23S, and inoculated with *Cmm*. After 1, 3, 5, and 7 days, the shoots were harvested and used for RNA extraction, and real-time qPCR. Genes analyzed are **(A)** *PR1a*, **(B)** *PI2*, and **(C)** *ACO*. Error bars indicate standard error of the mean. Association with different letters indicates statistical significance based on ANOVA followed by Bonferroni multiple comparison (*p* = 0.05).

Transcript levels of *PR1a*, *PI2*, and *ACO* were also examined when tomato plants treated with *Pseudomonas sp.* 23S by soil drench and inoculation of *Cmm* into the main stem by needle injection after 5 days (**Figures 6A–C**). Day 5 was chosen because the previous physiological experiment, described above, indicated that the disease severity was smallest when *Cmm* was inoculated after 5 days, rather than 3 or 7 days. The transcript level of *PR1a* was not different among treatments at day 1; however, at days 3 and 5, the *P+C* treatment resulted in significantly higher transcript levels than other treatments (54 fold at day 3 and 58 fold at day 5; **Figure 6A**). At day 7, its transcript level was still higher (55 fold), and the transcript levels for the *Pse* and *Cmm* treatments were also as high as that of the *P+C* treatment (34 fold for the *Pse* and 75 fold for *Cmm* treatment; **Figure 6A**). The transcript levels of *PI2* were relatively high at day 1 for all of the treatments (87, 25, 48, and 68 fold for the *Cont*, *Pse*, *Cmm* and *P+C* treatments, respectively) as compared with those at day 3, 5, 7 (**Figure 6B**). In addition, they were variable among biological replicates, resulting in large standard errors. No differences were detected among treatments at days 1, 3 and 5. At day 7, the transcript level of the *Cmm* treatment (ninefold greater than the control) was significantly higher than other treatments. The transcript level of *ACO* gradually increased going from days 1 to 7 (**Figure 6C**). For every time point: the transcript level of the *Cont* treatment was the lowest; the level of the *Pse* and *Cmm* treatments were similar to or slightly higher than those of the *Cont* treatment; and, no difference was detected between the *Pse* and *Cmm* treatments. The *P+C* treatment was always the highest among the treatments (1.5, 1.75, 2.5, and 4.5 fold relative to the *Cont*, *Pse*, *Cmm* and *P+C* treatments, respectively; **Figure 6C**).

## Discussion

*Pseudomonas* *sp*. 23S was shown to possess key PGPR traits, but its degree differed among traits. Specifically, production of siderophores and indole acetic acids were clearly demonstrated. Since siderophores facilitate iron acquisition, especially under iron-limited conditions, siderophore-production represents a biocontrol mechanism for suppression of root diseases by rhizobacteria (Schippers et al., 1987; O’Sullivan and O’Gara, 1992; Shanmugaiah et al., 2015). Similar to past findings (Idris et al., 2007), the amount of IAA produced by *Pseudomonas* *sp*. 23S was dependent on the concentration of the IAA precursor tryptophan. Bacteria-produced IAA is known to alter root architecture and support plant development (Dodd et al., 2010; Bhattacharyya and Jha, 2012). In addition, *Pseudomonas* *sp*. 23S inhibited the growth of *Pseudomonas syringae* pv. *tomato* DC 3000 that causes bacterial speck disease of tomato (*Solanum lycopersicum*) and *Arabidopsis thaliana* (Whalen et al., 1991), suggesting potential for *Pseudomonas* *sp*. 23S as a biocontrol agent for this disease. On the other hand, *Pseudomonas* *sp*. 23S may not be a very good phosphorous (P) solubilizer, as the amount of P solubilized was relatively low as compared to other phosphorus-solubilizing PGPR (Rahi et al., 2010). *Pseudomonas* *sp*. 23S was shown to be a moderate producer of hydrogen cyanide (HCN) a volatile, antibiotic, secondary metabolite, which can contribute to disease suppression by some biocontrol bacteria (Voisard et al., 1989; Defago and Haas, 1990; Haas and Keel, 2003). Since HCN is known to inhibit *Cmm* growth (Lanteigne et al., 2012), production of HCN may partly explain the antagonistic activity that *Pseudomonas* *sp*. 23S has against *Cmm.*

While *in vitro* assay revealed the potential of *Pseudomonas* *sp*. 23S as a PGPR, the plant assay demonstrated that *Pseudomonas* *sp*. 23S has plant-growth promoting effects on tomato seedlings. In this experiment, the substrate was probably not the source of nutrients for seedlings, or for *Pseudomonas* *sp*. 23S, because it was composed of a mix of sand and turface, and the seedlings were supplied with water only. *Pseudomonas* *sp*. 23S might have utilized the nutrients from root exudates and synthesized chemicals that might have growth-promoting effects on the tomato seedlings. Many rhizobacteria are known to convert the root exudate tryptophan to IAA (Frankenberger and Muhammad, 1995), and as the *in vitro* assay suggested that *Pseudomonas sp.* 23S was an IAA producer, this mechanism could explain the enhanced root development of the plants treated with *Pseudomonas sp.* 23S. *Pseudomonas* *sp*. 23S was also shown to be a good colonizer of tomato roots, as it colonized the roots and established in good numbers within 3 days, similar to previous reports (Lugtenberg et al., 1999; Yan et al., 2003). We also tried to isolate *Pseudomonas* *sp*. 23S from inside the root tissue by sterilizing the root surface, but we were not able to do so (data not shown). Hence, *Pseudomonas* sp. 23S seems to reside only on the root surface (rhizoplane) and in the soil around the root (rhizosphere), not the inside the root, and causes plant growth promotion.

*Pseudomonas sp.* 23S triggered ISR, which probably contributed to the reduction of bacterial canker severity by *Cmm* in tomato plants when *Cmm* was inoculated 5 days after the *Pseudomonas sp.* 23S application. In this study, *Pseudomonas sp.* 23S was applied before *Cmm* inoculation, and the two bacteria were spatially separated since the *Pseudomonas sp.* 23S was applied as soil drench and *Cmm* was injected to the main stem by syringe needle. We tested for the presence of *Pseudomonas sp*. 23S in the stem samples by using a selective plate assay, but the bacteria were never detected (data not shown). Thus, direct contact between *Cmm* of the *Pseudomonas sp.* 23S was not likely to occur, and the bacterial canker reduction that was observed must have been a result of ISR effects. *Cmm* can survive as an endophyte in tomato plants, but induction of disease symptoms requires a certain minimum population level, generally 10^8^ cfu g^−1^ of plant tissue (Gartemann et al., 2003). Compared to this number, as well as to the reports from past studies (Balaji et al., 2008; Sharabani et al., 2013), the *Cmm* population in the stem samples in our study was relatively small, although leaf wilting was clearly observed. This could be because the tomato plants used in our study were younger than those used in other studies (17–21 vs. 28 days). Since we observed the disease progression over 3 weeks, we cannot elaborate on the effects of *Pseudomonas sp.* 23S application on older plants or at later growth stages. However, most plants inoculated with *Cmm*, but without the *Pseudomonas sp.* 23S treatment, were severely infected and would not be able to recover as the main stems were completely broken down. Treatment with *Pseudomonas* *sp*. 23S reduced the *Cmm* population and limited the disease severity as indicated by the fact that these plants stood straight and their leaves were unwilted or less wilted. This effect is certainly important considering that the protection of plants at this stage is more critical than that of older plants that are more resistant to the disease.

In our experimental system, the age and/or size of the plants at the time of the *Cmm*-inoculation might have influenced the disease progression within plants. The date of visual symptom appearance, and the severity of bacterial canker are affected by temperature, plant age, inoculum concentration, and cultivar (Chang et al., 1992b). In young tomato plants, the disease symptoms caused by *Cmm* are known to appear earlier and they are more susceptible to infection than mature plants (Chang et al., 1992b; Sharabani et al., 2013). This may explain our observation that disease progression was relatively slow for the plants at the 7-day-interval. Nevertheless, at the plants at the 5-day interval the plants are younger than those at the 7-day interval and thus, the slower disease progression observed at this interval is most probably due to ISR, and not to an age-related resistance.

In our study, 5 days was the optimal interval between *Pseudomonas sp.* 23S application and *Cmm* inoculation, in terms of alleviating bacterial canker. Past studies showed that several days are required for ISR to develop and to deliver resistance against various phytopathogens (Babu et al., 2015). The different time interval between PGPR treatment and pathogen inoculation could be related to the population size of the PGPR. The protection by PGPR-mediated ISR is said to be apparent only when the roots were colonized by PGPR at a specific threshold population size (Raaijmakers et al., 1995). Also, Zhang et al. (2004) indicate “quorum sensing effects,” where a certain bacterial population density is essential to produce a signal molecule that is involved in provoking ISR.

The results showed that the *Pseudomonas sp.* 23S increased the transcript level of the *PR1a*, but not of *PI2* and *ACO*, suggesting that the ISR induced by the *Pseudomonas sp.* 23S may involve the salicylic acid (SA) pathway, rather than jasmonic acid (JA) or ethylene (ET). The *PR1a* gene codes for a pathogenesis-related protein, and has been used as a marker gene for salicylic acid resistance induction (Park et al., 2001; Block et al., 2005; Niu et al., 2012; Martínez-Medina et al., 2013). *PI2* gene codes for a proteinase inhibitor and is induced by wounding and jasmonic acid (JA; Peña-Cortés et al., 1995; Peiffer et al., 2009; Niu et al., 2012; Martínez-Medina et al., 2013). *ACO* is a gene coding for 1-aminocyclopropane-1-carboxylix acid (ACC) oxidase, the level of which is related to ethylene (ET) production since ACO is an enzyme that converts ACC into ethylene (Stearns and Glick, 2003; Yim et al., 2014). Although JA/ET are generally considered to be key hormone(s) for ISR response, which is mediated by non-pathogenic plant growth promoting bacteria (PGPR), different results have been reported from more recent studies (De Vleesschauwer and Höfte, 2009), and the ISR by the *Pseudomonas sp.* 23S seems to fall into this new trend. The signaling pathway for ISR seems species specific, that is specific to the rhizobacterium, and pathogen involved (Ryu et al., 2003; Djavaheri, 2007; Conn et al., 2008). Researchers agree that hormone crosstalk plays an important role in regulating ISR. Regarding SA-and JA/ET pathways, antagonistic interaction has been documented from many studies (Koornneef and Pieterse, 2008), and this might apply to our case, where *Pseudomonas sp.* 23S induced SA response but not JA and ET. The antagonistic interaction between SA and JA may be the outcome of resource allocation, costs of induction, or a means for the plant to adaptively tailor its responses to different enemies and a target for manipulation by enemies (Thaler et al., 2012). Generally, SA-dependent defense response is said to be effective against biotrophic pathogens, while JA/ET-dependent defense response is effective against necrotrophic pathogens (Sorokan et al., 2013). In this respect, *Cmm* would be a suitable target for the SA-dependent ISR because *Cmm* is considered as a biotrophic pathogen (Eichenlaub and Gartemann, 2011). We cannot exclude the possibility that JA and ET are not involved in the ISR provoked by the *Pseudomonas sp.* 23S. Mutant plants impaired in SA, JA, and ET pathways could be utilized to confirm whether these hormones are required for ISR.

Furthermore, the results demonstrated priming effect of the *Pseudomonas sp.* 23S treatment. Faster and/or greater response of defense-genes - priming has been observed for many ISR-inducing PGPR (Pieterse et al., 2014). Accumulation response of *PR1a* after *Cmm* inoculation was faster and quantitatively greater with the plant pre-treated with the *Pseudomonas sp.* 23S than the plants without the pre-treatment. Since the *Pseudomonas sp.* 23S treatment alone also induces accumulation response of *PR1a*, the prior- *Pseudomonas sp.* 23S treatment probably prepares the plants for the pathogen attack, by making this response faster and greater and enhancing the defense capacity of the plants. Priming may explain the disease reduction observed under the 5-day interval in our study. To understand the effects of the *Pseudomonas sp.* 23S treatment for disease reduction, studying the responses of other defense-related genes (e.g., other PR proteins, defense-related enzymes) and whether they do also show priming effects would be helpful.

Faster and greater accumulation response was also observed for the *ACO*, but the situation may be different from the *PR1*, because the *ACO* transcript abundance was not affected by *Pseudomonas sp.* 23S treatment alone. From past studies, ethylene is known to play a critical role in bacterial canker of tomato. Plants with reduced ethylene production or impairment of ethylene perception results in decrease in the disease severity (Balaji et al., 2008; Savidor et al., 2011), and thus host-derived ethylene is suggested to be a requirement for the disease development by *Cmm*. Our results that the *Pseudomonas sp.* 23S treatment alone did not significantly affect the *ACO* abundance but the same treatment did after *Cmm* inoculation supports the past studies in that ethylene is involved in the disease infection by *Cmm*. At the same time, the fact that *Pseudomonas sp.* 23S treatment can make this response faster and greater in quantitatively and alleviate the disease may suggest that the *Pseudomonas sp.* 23S might have modulated the role of ethylene. This consequently could influence the disease development by *Cmm* and thus might have contributed to the disease reduction as observed in our study.

The transcript level of *PI2* showed different trend from that of *PR1a* or *ACO*, elevated on day 1, especially for the control treatment. This may be due to the damaging nature of applying the mock (needle injection) as the transcript level decreased at later time points. For *Cmm* inoculated plants, however, its level remained high at day 7. Treatment with *Pseudomonas sp.* 23S might have explained this: while the *Cmm* inoculated plants must combat *Cmm* invasion, the same plants were less affected by the prior *Pseudomonas sp.* 23S treatment due to ISR effects.

In this study, we investigated *Pseudomonas sp*. 23S, which was previously isolated based on the *in vitro* antagonistic activity against *Cmm*. The characterization study of *Pseudomonas sp.* 23S revealed great potential of this strain in agriculture, both for plant growth promotion and as a biocontrol agent. Future study could investigate its effectiveness in field condition. This study also demonstrated that *Pseudomonas sp.* 23S could induce ISR in tomato plants and reduce the severity of tomato bacterial canker disease that is caused by *Cmm*. The best time interval between the *Pseudomonas sp.* 23S treatment and *Cmm* inoculation for reducing the severity of bacterial canker was 5-days in our experimental system, which used drench application of *Pseudomonas sp.*, stem inoculation of *Cmm*, and young tomato plants; this interval, as well as the effectiveness of ISR, could change with different methods, timing of bacterial application and of pathogen inoculation, and plant ages. Such information would be useful, especially for the commercial use of this bacterium in the future. Our study also suggested that the ISR by *Pseudomonas sp.* 23S may involve SA in its signaling pathway. However, the possibility of JA and/or ET involvement should not be ignored. Mutant plants with impaired hormonal pathways could be studied in the context of a *Cmm* infection to confirm their involvement. In addition, our results provided new insights on the role of ethylene in disease development of *Cmm*. Further studies to elucidate the signaling pathways of *Pseudomonas sp.* 23S ISR would certainly add knowledge for understanding molecular mechanism of ISR induced by PGPR but it would provide useful information regarding the disease strategies taken by *Cmm*.

## Author Contributions

YT wrote the manuscript, conducted the growth chamber experiments, gene expression studies, and analysis of the data. J-BC and DS guided and supervised the overall study.

## Conflict of Interest Statement

The authors declare that the research was conducted in the absence of any commercial or financial relationships that could be construed as a potential conflict of interest.

## Supplementary Material

FIGURE S1 Anti-*Cmm* activity of *Pseudomonas sp*. 23S *in vitro*. One hundred micro liter of *Cmm* culture was spread on Nutrient Broth Yeast Extract (NBYE) agar. A sterile filter-paper-disk (6 mm diameter) was placed on the agar surface. Five micro liter of the newly isolated bacterium culture (on the right), and Nutrient Broth (on the left) were applied on the respective disks. The plate was sealed with parafilm and incubated at 28°C for 2 days. Zone of inhibition = 5 mm.

FIGURE S2 Characterization of *Pseudomonas sp.* 23S for PGPR traits. **1** **(a)** Phosphorus solubilization on a PVK, NB media (top), *Pseudomonas sp.* 23S (left and right), and positive control bacterium (bottom). **1** **(b)** Phosphorus solubilization on a PBRIP agar plate, NB media (left), and of *Pseudomonas sp.* 23S (right). **2** Siderophore production, NB media (left), and *Pseudomonas sp.* 23S culture (right). **3** Hydrogen cyanide production, **(a)** Kings B media, **(b)** *Pseudomonas sp*. 23S, and **(c)** a positive control bacterium. **4** Antagonistic activities against *Pseudomonas syringae* pv. *tomato* DC3000.

FIGURE S3 Representive plant photos showing the effects of *Pseudomonas sp*. 23S treatment 5-day prior to *Cmm* inoculation. Two-week-old tomato plants were treated with *Pseudomonas sp.* 23S by soil drench, and after 5 days, *Cmm* was inoculated in the main stem by needle injection: **(A)** *Cont*, control; **(B)** *Pse*, treated with *Pseudomonas sp.* 23S; **(C)** *Cmm*, inoculated with *Cmm*; and **(D)** *P+C*, treated with *Pseudomonas sp.* 23S, and inoculated with *Cmm.*

FIGURE S4 Area under disease progression curve (AUDPC). Two-week-old tomato plants were treated with *Pseudomonas sp.* 23S by soil drench, and after 5 days, *Cmm* was inoculated in the main stem by needle injection. The AUDPC was based on the percentage of wilted leaves during 3 weeks after the date of *Cmm* inoculation (presented by **Figure 2** in the main text): *Cmm*, inoculated with *Cmm*; and *P+C*, treated with *Pseudomonas sp.* 23S, and inoculated with *Cmm*. Error bars indicate standard error of the mean. An asterisk indicates significant difference from the *Cmm* treatment after ANOVA followed by Tukey’s multiple comparison test (*n* = 14, *p* = 0.05).

FIGURE S5 Effects of *Pseudomonas sp.* 23S treatment 5 days prior to *Cmm* inoculation on **(A)** plant height, and **(B)** leaf area. Two-week-old tomato plants were treated with *Pseudomonas sp.* 23S (or 10 mM MgSO_4_) by soil drench, and after 3, 5, or 7 days, *Cmm* (or 10 mM MgSO_4_) was inoculated into the main stem by needle injection. The plant height and leaf areas were measured. Error bars indicate standard error of the mean. Association with different letters indicate statistical significance based on ANOVA followed by Tukey’s multiple comparison test. Treatments are: *Cont*, control; *Pse*, treated with *Pseudomonas sp.* 23S; *Cmm*, inoculated with *Cmm*; and, *P+C*, treated with *Pseudomonas sp.* 23S, and inoculated with *Cmm* (*n* = 14, *p* = 0.05).
