# Supplementary figures and images for: Biocontrol Rhizobacterium Pseudomonas sp. 23S Induces Systemic Resistance in Tomato (Solanum lycopersicum L.) Against Bacterial Canker Clavibacter michiganensis subsp. michiganensis
Source: Front Microbiol. 2018 Sep 11;9:2119. doi: 10.3389/fmicb.2018.02119 (PMC6141633; doi:10.3389/fmicb.2018.02119)

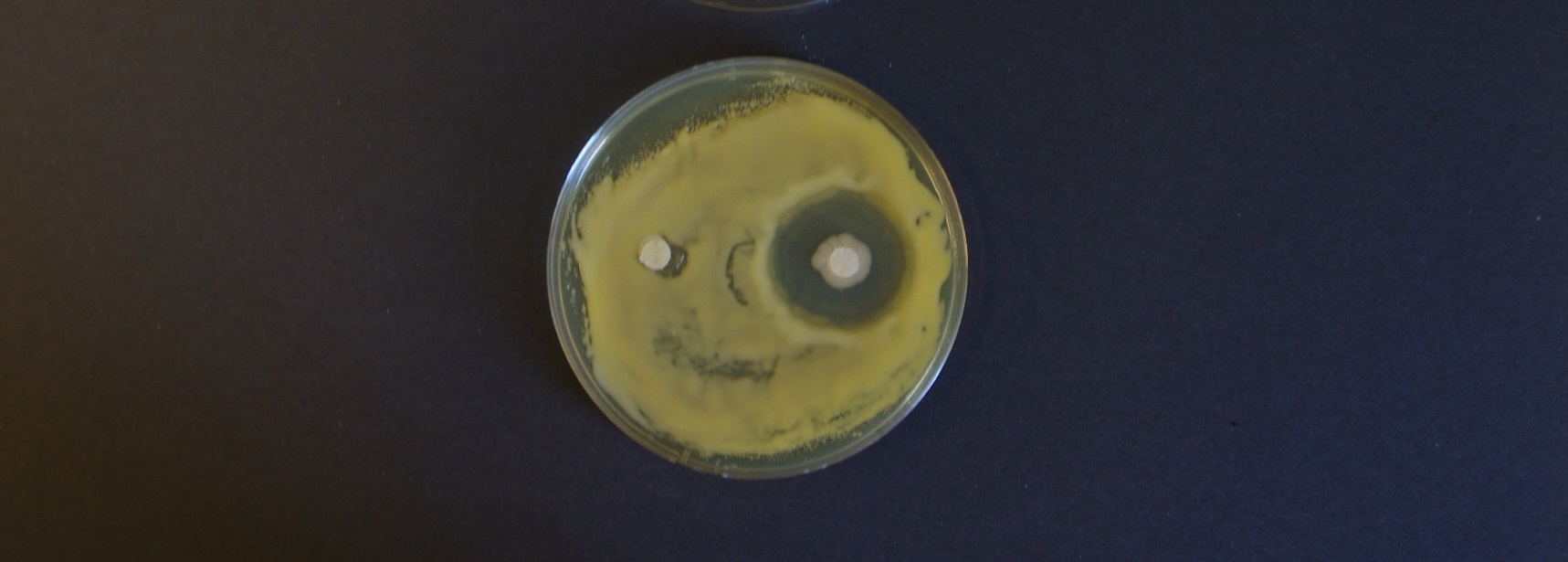

Supplement: FIGURE S1 — Anti-Cmm activity of Pseudomonas sp. 23S in vitro. One hundred micro liter of Cmm culture was spread on Nutrient Broth Yeast Extract (NBYE) agar. A sterile filter-paper-disk (6 mm diameter) was placed on the agar surface. Five micro liter of the newly isolated bacterium culture (on the right), and Nutrient Broth (on the left) were applied on the respective disks. The plate was sealed with parafilm and incubated at 28°C for 2 days. Zone of inhibition = 5 mm. [file Image_1.JPEG]

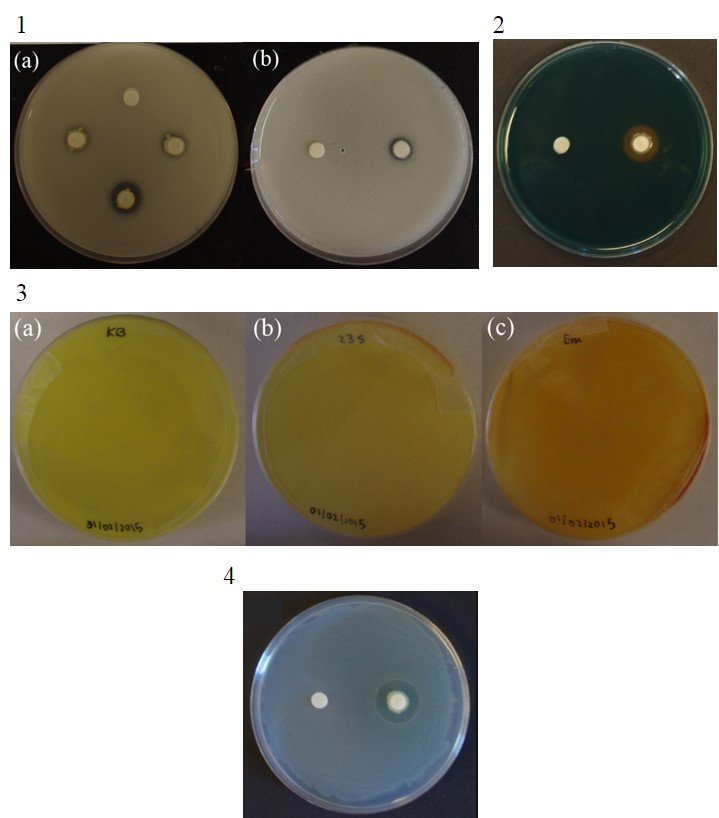

Supplement: FIGURE S2 — Characterization of Pseudomonas sp. 23S for PGPR traits. 1 (a) Phosphorus solubilization on a PVK, NB media (top), Pseudomonas sp. 23S (left and right), and positive control bacterium (bottom). 1 (b) Phosphorus solubilization on a PBRIP agar plate, NB media (left), and of Pseudomonas sp. 23S (right). 2 Siderophore production, NB media (left), and Pseudomonas sp. 23S culture (right). 3 Hydrogen cyanide production, (a) Kings B media, (b) Pseudomonas sp. 23S, and (c) a positive control bacterium. 4 Antagonistic activities against Pseudomonas syringae pv. tomato DC3000. [file Image_2.JPEG]

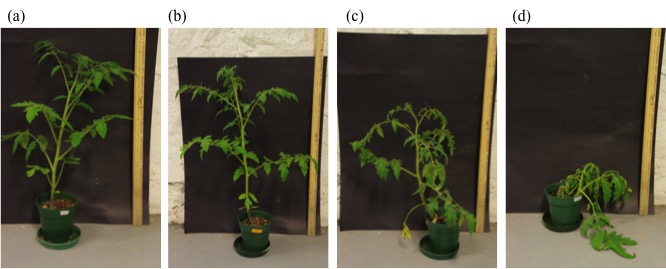

Supplement: FIGURE S3 — Representive plant photos showing the effects of Pseudomonas sp. 23S treatment 5-day prior to Cmm inoculation. Two-week-old tomato plants were treated with Pseudomonas sp. 23S by soil drench, and after 5 days, Cmm was inoculated in the main stem by needle injection: (A) Cont, control; (B) Pse, treated with Pseudomonas sp. 23S; (C) Cmm, inoculated with Cmm; and (D) P+C, treated with Pseudomonas sp. 23S, and inoculated with Cmm. [file Image_3.JPEG]

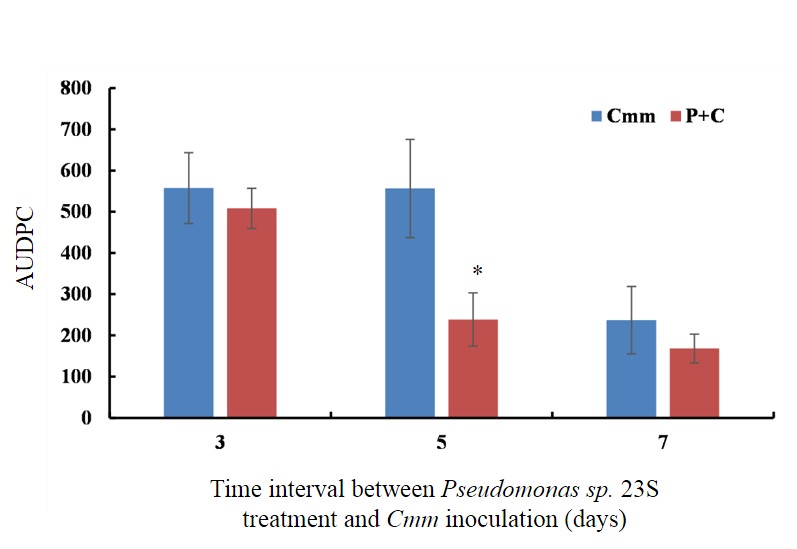

Supplement: FIGURE S4 — Area under disease progression curve (AUDPC). Two-week-old tomato plants were treated with Pseudomonas sp. 23S by soil drench, and after 5 days, Cmm was inoculated in the main stem by needle injection. The AUDPC was based on the percentage of wilted leaves during 3 weeks after the date of Cmm inoculation (presented by Figure 2 in the main text): Cmm, inoculated with Cmm; and P+C, treated with Pseudomonas sp. 23S, and inoculated with Cmm. Error bars indicate standard error of the mean. An asterisk indicates significant difference from the Cmm treatment after ANOVA followed by Tukey’s multiple comparison test (n = 14, p = 0.05). [file Image_4.JPEG]

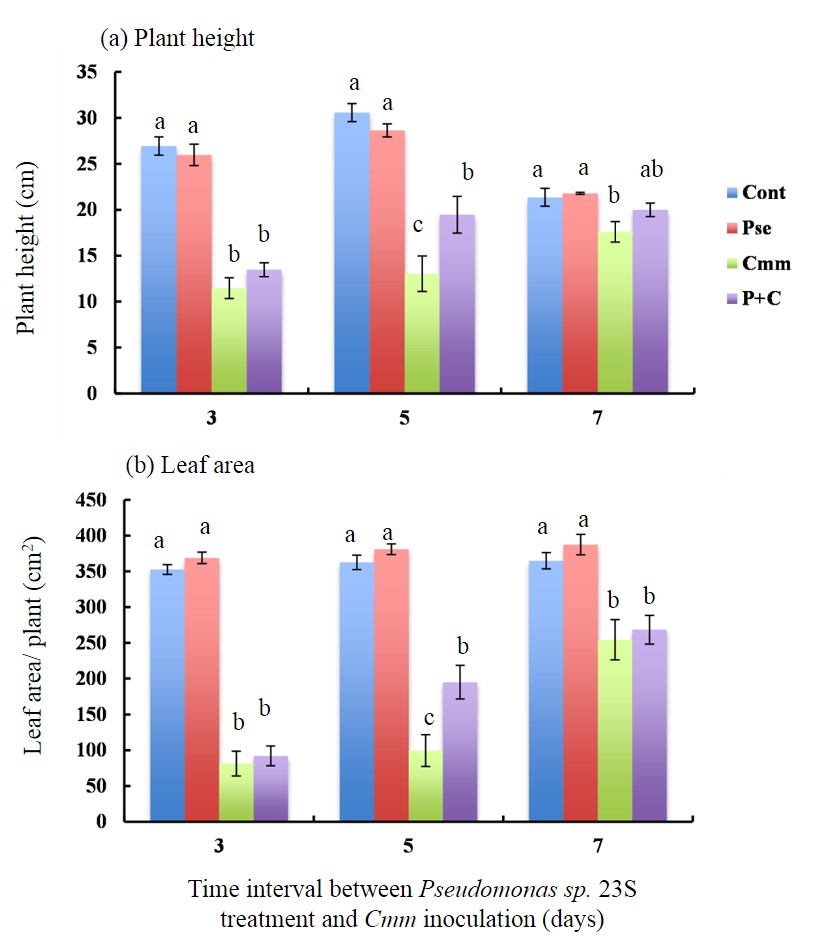

Supplement: FIGURE S5 — Effects of Pseudomonas sp. 23S treatment 5 days prior to Cmm inoculation on (A) plant height, and (B) leaf area. Two-week-old tomato plants were treated with Pseudomonas sp. 23S (or 10 mM MgSO4) by soil drench, and after 3, 5, or 7 days, Cmm (or 10 mM MgSO4) was inoculated into the main stem by needle injection. The plant height and leaf areas were measured. Error bars indicate standard error of the mean. Association with different letters indicate statistical significance based on ANOVA followed by Tukey’s multiple comparison test. Treatments are: Cont, control; Pse, treated with Pseudomonas sp. 23S; Cmm, inoculated with Cmm; and, P+C, treated with Pseudomonas sp. 23S, and inoculated with Cmm (n = 14, p = 0.05). [file Image_5.JPEG]
